# Wet chemical synthesis and magnetic properties of single crystal Co nanochains with surface amorphous passivation Co layers

**DOI:** 10.1186/1556-276X-6-285

**Published:** 2011-04-04

**Authors:** Shao-Min Zhou, Shi-Yun Lou, Yong-Qiang Wang, Xi-Liang Chen, Li-Sheng Liu, Hong-Lei Yuan

**Affiliations:** 1 Key Lab for Special Functional Materials of Ministry of Education, Henan University, 475004 Kaifeng, People's Republic of China

## Abstract

In this study, for the first time, high-yield chain-like one-dimensional (1D) Co nanostructures without any impurity have been produced by means of a solution dispersion approach under permanent-magnet. Size, morphology, component, and structure of the as-made samples have been confirmed by several techniques, and nanochains (NCs) with diameter of approximately 60 nm consisting of single-crystalline Co and amorphous Co-capped layer (about 3 nm) have been materialized. The as-synthesized Co samples do not include any other adulterants. The high-quality NC growth mechanism is proposed to be driven by magnetostatic interaction because NC can be reorganized under a weak magnetic field. Room-temperature-enhanced coercivity of NCs was observed, which is considered to have potential applications in spin filtering, high density magnetic recording, and nanosensors.

**PACS:** 61.46.Df; 75.50; 81.07.Vb; 81.07.

## Introduction

In the last decade, diverse technological applications of magnetic nanostructures in magnetofluid, recording tape, flexible disk recording media, permanent magnets, microwave oscillators as well as biomedical materials, and catalysts have provided an impetus for extensive research in nanometer scale magnets [1-17]. Most of these applications rely on the stability of ferromagnetic ordering with temperature. In nanometer scale magnets, the thermal fluctuations randomize the magnetic moment by overcoming the anisotropy energy leading to unstable state of paramagnetism (non-magnetic materials) or superparamagnetism (corresponding coercivity and hysteresis fall to zero). Cobalt (Co) is superior to other ferromagnetic materials because of its highest Curie temperature (*T*_c_) of about 1394 K, which is crucial for thermal stability in high-temperature nanodevice applications [1,5-16]. For realizing high *T*_c_, magnetization, and coercivity, the aim must be directed toward increasing the amount of the ferromagnetic Co phase [7-10]. Owing to its basic metallic characteristic, pure cobalt, especially for nanosized Co, is very reactive and must be unstabilized in ambient air [11], and therefore, its use has been limited to prepare Co nanostructures in the absence of the shell [11-16]. A very simple bottom-up method is to produce stable film-assisted synthesis for a surface with slightly controlled Co to passivate the surface of the host materials, including organic and inorganic templates, and alloyed technique [1,2,5-10,12-16]. For example, recently, control of magnetism in cobalt nanoparticles by oxygen passivation was reported by Srikala's research group [14,15], and cobalt nanowires with controlled diameters have been synthesized using electrochemical deposition in etched-ion-track polycarbonate membranes [16]. In this latter case, however, corresponding magnetic properties are inevitably decreased by the addition of non-magnetic materials or natural oxide layers [1,5-16]. As far as we know, amorphous phases lack long-range crystalline order and have unique electronic, magnetic and corrosion-resistant properties. In this article, based on our earlier study [17], we report for the first time a wet chemical synthesis of high-pure Co nanochains (NCs) without any oxide shells and templates. The amorphous Co covering layer would be able to protect the active Co core from oxygen in atmosphere. In particular, the room-temperature coercivity (up to 355.8 Oe) of the NCs is larger than that (93.6 Oe) of pure single-crystal Co (PSC) metal, which will make them as promising candidates for advanced magnetic media and investigative studies on novel great magnetoresistive properties.

## Experimental section

In a typical experiment, 10.0 mL glycerine was heated to the boiling point (approx 560 K) and refluxed for 3 min. Then, 50 mL of hydrazine monohydrate was added dropwise to the boiling solution. After 1 min, 10.0 mL of Co (NO_3_)_2 _6H_2_O solution (0.5 mol/L, in glycerine) and 10.0 mL of hydrazine hydrate solution (0.5 mol/L, in glycerine) were added rapidly to the boiling solution under vigorous magnetic stirring. After refluxing for approx 80 min, as proposed in our previous article [17], the final products in the form of loose powders (large quantities of light-gray wool-like products) were obtained by centrifugation under permanent-magnet (approx. 0.5 T). The powders were rinsed repeatedly with absolute ethanol for several times, followed by the removal of the residual solvent through evaporation in vacuum at 500 K. The yield of the as-prepared Co specimens is about 60% according to our calculation. We noticed that no sign of oxidation was observed on the as-synthesized metal Co NCs even after aging for over 1 month under ambient conditions. This indicates that the Co NCs are very stable after surface modification with amorphous Co, which is very important for future applications. The samples were characterized extensively for morphology, phase, and chemical composition using scanning/transmission electron microscopy (SEM/TEM), energy dispersive X-ray spectroscopy (EDS), X-ray powder diffraction (XRD), selective area electron diffraction (SAED), high resolution TEM (HRTEM), and X-ray photoelectron spectroscopy (XPS). The temperature dependence of magnetization and room temperature (RT) hysteresis curves were carried out by means of vibrating sample magnetometer (VSM, Model 4 HF) and physical properties measurement system (PPMS, Quantum Design PPMS-7).

## Results and discussion

**Figure 1:**
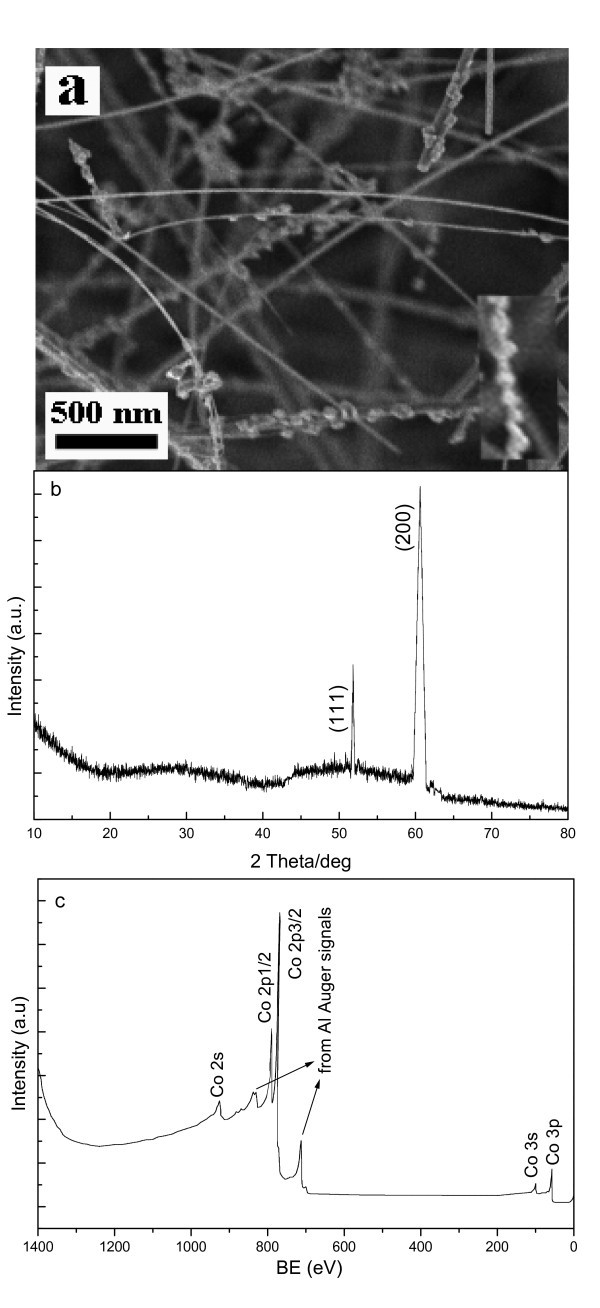
**A typical (a) SEM image (inset: HRSEM), (b) XRD and (c) XPS patterns of the collected Co-NCs**.

**Figure 2:**
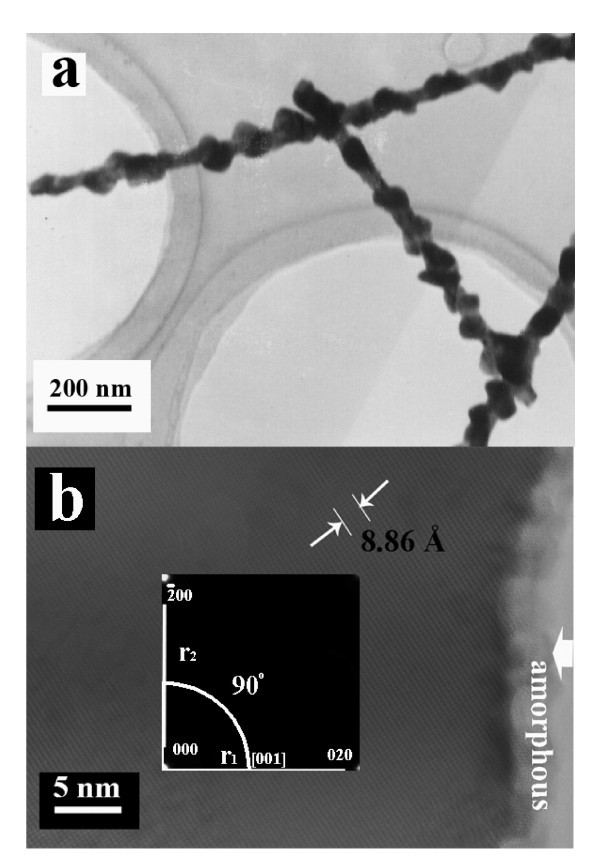
**A typical (a) TEM and (b) HRTEM image of Co-NCs (inset: SAED pattern)**.

**Figure 3:**
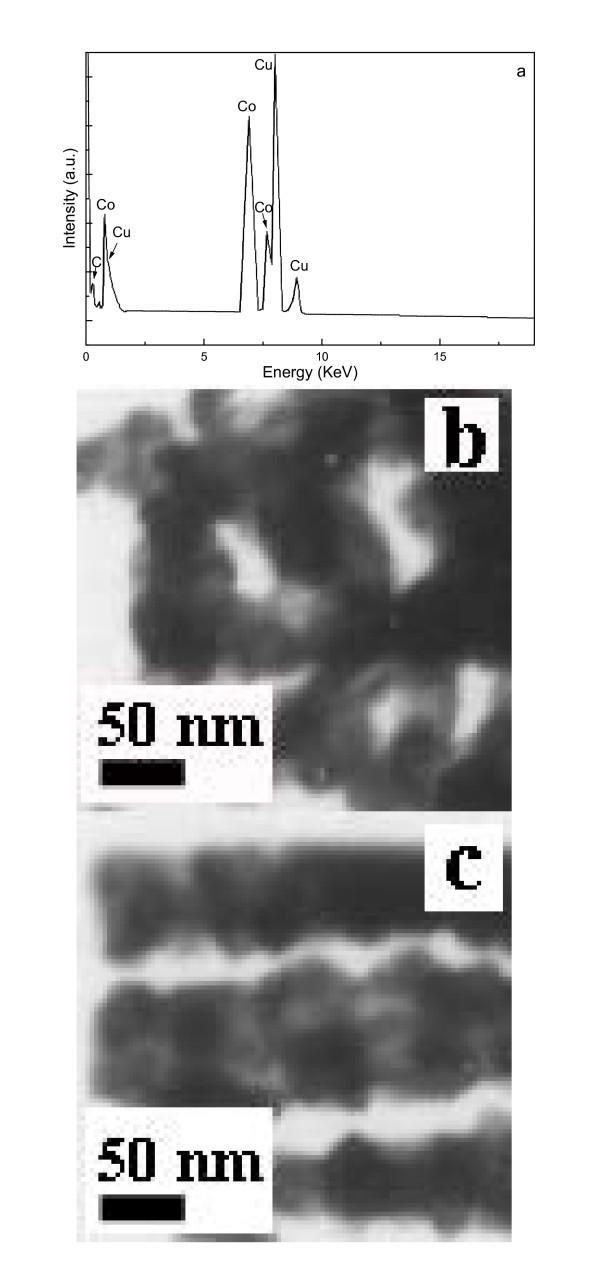
**(a) EDS pattern of the as-synthesized NCs, and both TEM images of the (b) nonaligned and (c) aligned nanoparticles, respectively, obtained from without external magnetic field and with weak external magnetic field (approx. 0.5 T)**.

Figure 1a shows a typical SEM image, in which high dense (high-yield) chain-like nanostructures are observed. One can observe that the diameter and length of single NCs are approx. 60 nm and several micrometers, respectively. A high-resolution SEM image is demonstrated in the inset of Figure 1a where a nanoparticle array, taken from a single NC, is clearly seen. Based on SEM statistic analysis, the yield of the NCs is for approx. 60%. The XRD patterns of bulk Co NCs (Figure 1b) reveal that the two sharp diffraction peaks can be assigned to the Co face-centered cubic (FCC/*Fm*3*m*) structure (*a *= 0.354 nm) (see Card No. 15-0806, JCPDS-ICDD, June 2002). Two very broad peaks are noticed in 2 theta angles (20-40 and 45-60), which may result from the amorphous passivation Co layers and substrate. In addition, no impurity phases such as cobalt oxides or precursor compounds have been confirmed within instrumental error. More accurately, XPS was used to determine the composition of the bulk Co-NC samples. As shown Figure 1c, a range of XPS spectrum is indicated, in which the intensive peaks located at 778.3 eV (Co2p3/2) and 793.3 eV (Co2p1/2) correspond to the respective electronic states of metallic Co. As indicated with the arrows, these wide peaks (from 831 to 838 and from 777 to 698) originate from auger lines of monochromated Al for XPS characterization. It is clear that the NCs are high-pure Co^0 ^with other elements absent, which is in very good agreement with the results of XRD. As shown in Figure 2a, a typical slight enlargement TEM image of the as-synthesized large-scale NCs is exhibited. From the TEM image, one can, by closer observation, conclude that perfectly aligned nanoparticles (chain-like nanostructures) were produced and the diameter of individual Co NCs is approx. 60 nm. For microanalysis, HRTEM, SAED, and EDS were employed for phase, and composition of single NCs. A HRTEM image taken from a single nanoparticle is shown in Figure 2b, in which the presence of the gray edge without any stripes and the center with perfect continuous lattice fringes reveals the amorphous passivation shell (marked with the large white arrow) and high-quality crystal core growth. The lattice spacing of 8.86 Å (5 × 1.772 Å) is consistent with that of the [200] planes of single-crystalline face-centered Co, which is compatible with the data of XRD. The SAED patterns (see the inset in Figure 2b) are composed of the regular, clear diffraction dots, which reveal the single crystalline nature and can be indexed to the FCC Co. Diffraction patterns taken from different parts along the NC axis show the same features, indicating the same periodical orientation along the single-crystalline Co NC. Based on micro-composition-analysis, Figure 3a gives a typical EDS pattern, in which only the Co peaks can be indexed within experimental error, and the peaks of elements Cu and C are attributable to copper grid with carbon film.

The synthesized NCs can be reorganized in a weak magnetic field. Typically, the purified Co NCs were dispersed into de-ionized water by ultrasonic agitation. A drop of the Co NCs solution was dripped on a copper grid with holes and carbon film to characterize the TEM in the absence or the presence of the weak external magnetic field (about 0.5 T) and dried naturally. The result reveals that the NCs in the weak magnetic field have aligned according to the direction of the magnetic field as shown in Figure 3b, whereas without the external magnetic field, nonaligned NCs appear as shown in Figure 3c. Regarding the mechanism for the growth of the highly branched Co nanoparticle chains, we believe that magnetostatic interaction plays an important role. The magnetic dipole-dipole interaction displayed behavior similar to that of soft templates. Initially, very small Co nanoparticles were formed. With the increase of the growth time, presumably, the small Co nanoparticles diffused and aggregated to form larger nanoparticles. The Co nanoparticles were then assembled into neck-like chains with multiple branches because of the stronger anisotropic magnetic forces, and these findings are in agreement with those of our earlier article [17].

**Figure 4:**
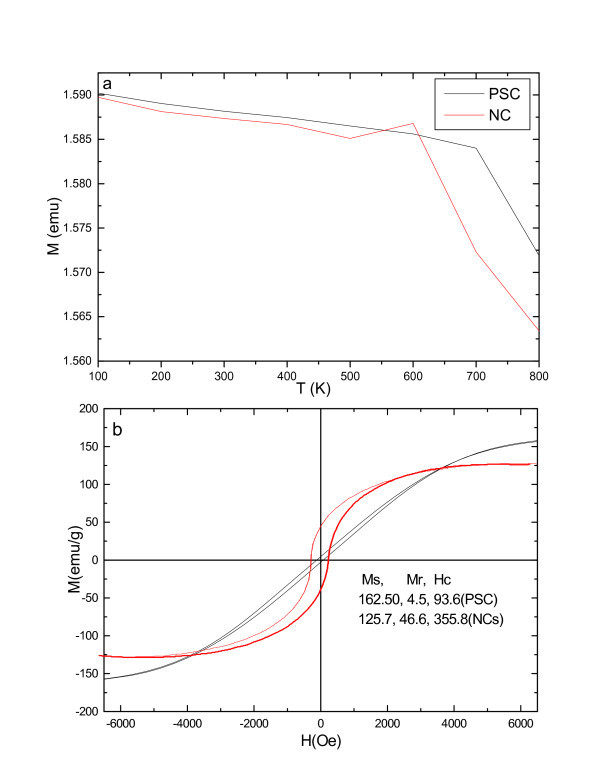
**(a) Magnetization versus temperature for Co NCs (red) and PSC (black) and (b) RT magnetic hysteresis curves of the Co NCs (red) and PSC (black)**.

Despite the presence of the amorphous buffer layer, the NCs display a strong ferromagnetic behavior. Figure 4a shows the magnetization curve as a function of temperature for the NCs and PSC metal. As can be seen, for the NCs, a sharp magnetic transition is observed for *T *= ~600 K as determined from the inflection point of the magnetization versus temperature curve; the inflection point may result from the enhanced single-crystal Co mass from amorphous Co because the corresponding magnetic transition disappears in PSC under the same conditions. Figure 4b shows the magnetic hysteresis loop, measured at RT with the applied magnetic field perpendicular to the substrate surface. Based on both these curves, coercive fields (*H*_c_) of 355.8 and 93.6 Oe, respectively, for NCs and PSC, saturation magnetizations (*M*_s_) of 125.7 and 162.5 emu/g, respectively, for NCs and PSC, and remnant magnetization (*M*_r_) up to 46.6, and 4.5 emu/g, respectively, for NCs and PSC were determined. A detailed analysis of the magnetic properties as a function of the NC with various amorphous shell sizes will be published separately.

## Conclusion

In this study, this proposed method provides a simple and inexpensive method for the preparation of stable, magnetic Co NCs with the complete absence of impurities. The as-synthesized NCs are produced with increasing *H*_c _and *M*_r _at room temperature. In addition, the large *H*_c_, coupled with the controllable coercive field, makes these NC arrays the preferable candidates for probe-based data-storage systems. Introducing long-range, 1D translational order over macroscopic distances among the NCs will undoubtedly be a key driver in this respect, and is an area that clearly warrants future exploration.

## Abbreviations

EDS: energy dispersive X-ray spectroscopy; HRTEM: high-resolution TEM; NCs: nanochains; PSC: pure single-crystal Co; SAED: selective area electron diffraction; SEM: scanning electron microscopy; transmission electron microscopy (TEM); XPS: X-ray photoelectron spectroscopy; XRD: X-ray powder diffraction; RT: room-temperature; VSM: vibrating sample magnetometer; PPMS: physical properties measurement system;

## Competing interests

The authors declare that they have no competing interests.

## Authors' contributions

SM: carried out the experimental and numerical calculations, as well as drafted the manuscript. VSM and PPMS: performed an analysis and interpretation of results, and gave final approval of the version to be published. SY and YQ: partly carried out the TEM and HRTEM experiments, as well as drafted the manuscript. XL, LS, and HL: conceived of the study, and participated in its design and coordination. All authors read and approved the final manuscript.

## References

[B1] Whitney T Searson P Jiang J Chien C Fabrication and magnetic-properties of arrays of metallic nanowires Science 1993 261 1316 10.1126/science.261.5126.1316 17731862

[B2] Li P Wang R Chen W Chen C Gao X Wee A Well-aligned nickel nanochains synthesized by a template-free route Nanoscale Res Lett 2009 4 480 10.1007/s11671-009-9260-7 20672141 PMC2893928

[B3] Zhou S Liu L Yuan H Chen X Lou S Hao Y Yuan R Li N Magnetic properties of Ni-doped ZnO nanocombs by CVD approach Nanoscale Res Lett 2010 5 1284 10.1007/s11671-010-9639-5 20676203 PMC2897038

[B4] Yuan H Wang Y Zhou S Liu L Chen X Lou S Yuan R Hao Y Li N Low-temperature preparation of superparamagnetic CoFe2O4 microspheres with high saturation magnetization Nanoscale Res Lett 2010 5 1718 10.1007/s11671-010-9718-7 PMC2964483 21124634

[B5] Cao H Xu Z Sang H Sheng D Tie C Template synthesis and magnetic behavior of an array of cobalt nanowires encapsulated in polyaniline nanotubules Adv Mater 2001 13 121 10.1002/1521-4095(200101)13:2<121::AID-ADMA121>3.0.CO;2-L

[B6] Knez M Bittner A Boes F Wege C Jeske H Maiss E Kern K Biotemplate synthesis of 3 nm nickel and cobalt nanowires Nano Lett 2003 3 1079 10.1021/nl0342545

[B7] Rohart S Raufast C Favre L Bernstein E Bonet E Dupuis V Magnetic anisotropy of CoxPt1-x clusters embedded in a matrix: Influences of the cluster chemical composition and the matrix nature Phys Rev B 2006 74 104408 10.1103/PhysRevB.74.104408

[B8] Zhang L Lan T Wang J Wei L Yang Z Zhang Y Template-free synthesis of one-dimensional cobalt nanostructures by hydrazine reduction route Nanoscale Res Lett 2011 6 68 10.1186/1556-276X-6-89 PMC3212205 27502680

[B9] Wang G Zhang F Zuo H Yu Z Ge S Fabrication and magnetic Properties of Fe65Co35-ZnO nano-granular films Nanoscale Res Lett 2010 5 1107 10.1007/s11671-010-9609-y 20596322 PMC2894252

[B10] Brands M Hassel C Carl A Electron-electron interaction in quasi-one-dimensional cobalt nanowires capped with platinum: Low-temperature magnetoresistance measurements Phys Rev B 2006 74 033406 10.1103/PhysRevB.74.033406

[B11] Li X Xu C Han X Qiao L Wang T Li F Synthesis and Magnetic Properties of nearly monodisperse CoFe2O4 nanoparticles through a simple hydrothermal condition Nanoscale Res Lett 2010 5 1039 10.1007/s11671-010-9599-9 20672131 PMC2893895

[B12] Narayanan T Shaijumon M Ajayan P Anantharaman M Synthesis of high coercivity core-shell nanorods based on nickel and cobalt and their magnetic properties Nanoscale Res Lett 2010 5 164 10.1007/s11671-009-9459-7 PMC2893701 20651915

[B13] Gangopadhyay S Hadjipanayis G Dale B Sorensen C Klabunde K Papaefthymiou V Kostikas A Magnetic properties of ultrafine iron particles Phys Rev B 1992 45 9778 10.1103/PhysRevB.45.9778 10000866

[B14] Srikala D Singh V Banerjee A Mehta B Patnaik S Control of magnetism in cobalt nanoparticles by oxygen passivation J Phys Chem C 2008 112 13882 10.1021/jp804086m

[B15] Srikala D Singh V Banerjee A Mehta B Patnaik S Synthesis and characterization of ferromagnetic cobalt nanospheres, nanodiscs and nanocubes J Nanosci Nanotechnol 2009 9 5627 10.1166/jnn.2009.1157 19928277

[B16] Maaz K Karim S Usman M Mumtaz A Liu J Duan J Maqbool M Effect of crystallographic texture on magnetic characteristics of cobalt nanowires Nanoscale Res Lett 2010 5 1111 10.1007/s11671-010-9610-5 20596344 PMC2894180

[B17] Zhou S Zhang X Gong H Zhang B Wu Z Du Z Wu S Magnetic enhancement of pure gamma Fe2O3 nanochains by chemical vapor deposition J Phys Condens Matter 2008 20 075217 10.1088/0953-8984/20/7/075217

